# Population Pharmacokinetics of Dasatinib in Healthy Subjects

**DOI:** 10.3390/ph17060671

**Published:** 2024-05-23

**Authors:** Walaa B. Hassouneh, Mutasim A. Al-Ghazawi, Mohammad I. Saleh, Naji Najib

**Affiliations:** 1 Department of Biopharmaceutics and Clinical Pharmacy, School of Pharmacy, The University of Jordan, Amman 11942, Jordan; whassouneh.933@gmail.com (W.B.H.); moh.saleh@ju.edu.jo (M.I.S.); 2 International Pharmaceutical Research Center, Amman 11196, Jordan; n_najib@iprc.com.jo

**Keywords:** dasatinib, population pharmacokinetics, body mass index, absorption rate constant, obesity, overweight, dose adjustment

## Abstract

Background and Objectives: Dasatinib is one of the tyrosine kinase inhibitors. The main use of these agents is inhibition of cancerous cell proliferation. The therapeutic importance of tyrosine kinase inhibitors raises the necessity of many types of investigations, especially the pharmacokinetic analysis of these drugs in humans. This analysis, along with other investigations and clinical research, will contribute to the overall knowledge of the drug. This study focused on the population pharmacokinetics of dasatinib. The objective of the study was to investigate the sources of the variability of dasatinib in a population pharmacokinetics study in healthy participants. Methods: We utilized 4180 plasma observations from 110 subjects who were administered SPRYCEL^®^ on two separate occasions under fasting conditions; data from 20% of the subjects (22 subjects) were extracted for the purpose of internal model evaluation and data from 88 subjects were used in modeling. The model was evaluated by visual predictive check of three different datasets. A two-compartmental model with first order absorption and transit compartment was considered the simplest base model to describe the data based on the corrected Bayesian information criterion evaluation. Covariates were tested through conditional sampling for the stepwise approach-screening procedure in Monolix 2020R1 version. Conditional sampling for the stepwise approach was used to include the correlated covariates within the base model in the forward inclusion step and then to eliminate them backwardly to ensure that the key covariates were kept in the model at the final stage. Results: The effect of body mass index on the absorption rate constant was considered as significant covariate in the final established model. Visual predictive check for simulations, 20% of the original dataset (internal dataset) and an external dataset demonstrated the appropriateness of the final model. Conclusions: Population pharmacokinetic modeling was performed to describe dasatinib pharmacokinetics in healthy subjects. Body mass index was considered as a factor that might be used in the future along with studies on patients to adjust the dosing regimens. Key Points: Dasatinib is classified as a highly variable drug; this variability was demonstrated in the study by the effect of body mass index on the absorption rate constant.

## 1. Introduction

**Figure 1:**
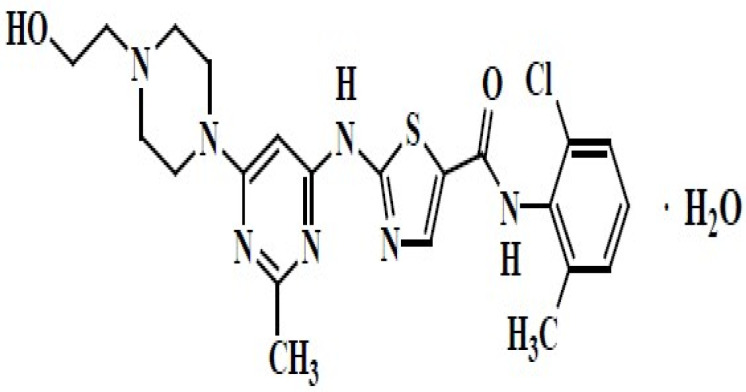
The chemical structure of dasatinib [4].

Dasatinib is one of the tyrosine kinase inhibitors (TKIs). It is indicated for the treatment of chronic myeloid leukemia (CML) and for acute lymphoblastic leukemia (ALL) with Philadelphia chromosome-positive (Ph^+^) in both adult patients and children [1,2,3]. SPRYCEL^®^ is the brand name of dasatinib available in various markets as tablets for oral use with different strengths, such as 20 mg, 50 mg, 70 mg, 80 mg, 100 mg, and 140 mg. It is also available as an oral suspension [3,4]. Dasatinib’s chemical name is N-(2-chloro-6-methylphenyl)-2-[[6-[4-(2-hydroxyethyl)-1-piperazinyl]-2-methyl-4-pyrimidinyl]amino]-5-thiazolecarboxamide monohydrate. C_22_H_26_ClN_7_O_2_S H_2_O is the molecular formula. The anhydrous free base has a molecular weight of 488.01 g/mol. The drug substance is insoluble in water and slightly soluble in ethanol and methanol [4]. It has three acid dissociation constant (pka) values (3.1, 6.8 and 10.8) [5]. The chemical structure of dasatinib is presented in Figure 1.

**Figure 2:**
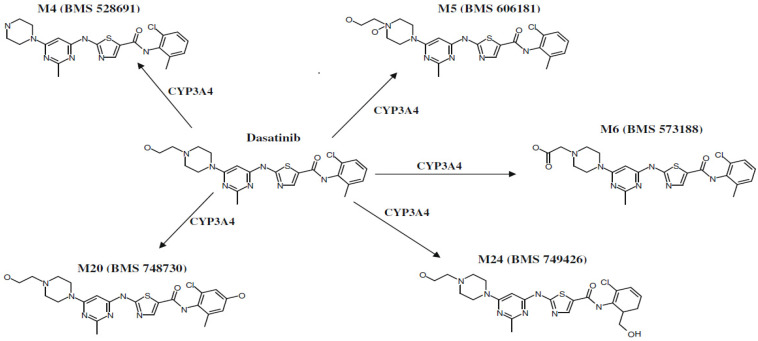
Schematic major metabolism of dasatinib [2].

Dasatinib can be classified as BCS class II (low solubility and high permeability) [3]. The pH variety in the gastrointestinal tract (GIT) fluids affects the solubility of dasatinib because of its weak basic nature. At a pH greater than 4, dasatinib’s solubility declines. Additionally, food, in principle, can affect dasatinib’s solubility by altering the gastric pH, so patients who take dasatinib with meals (high fat content) showed a clinically insignificant increase in the area under the curve (AUC). Generally, dasatinib has an adequate oral absorption, which is not affected by food [2,3]. Dasatinib reaches peak plasma concentrations within a relatively short period of time of 0.50–6.00 h [2,4]. Dasatinib has a large and variable apparent volume of distribution (2505 L with 93% CV) and is also bound extensively to proteins (96% of dasatinib is bound to human plasma in vitro). Dasatinib cannot reach the central nervous system (CNS) or cross the cerebrospinal fluid (CSF) barrier but some reports indicated that very low concentrations were detected in intracranial tumors; it is also a substrate of P-glycoprotein (P-gp) and breast cancer resistance protein (BCRP) [3,4,5]. Cytochrome P3A4 (CYP3A4) is the main enzymatic system from the CYP450 family that is responsible for dasatinib’s metabolism; it is metabolized into active and inactive metabolites, such as M4, M5, M6, M20 and M24 [2,3,4]. The schematic major metabolism of dasatinib is shown in Figure 2. The activity of dasatinib is not affected by its metabolites, although M4 showed some anti-proliferative activity in vitro [3]: M4 exhibited similar potency to dasatinib but with an insignificant effect on the parent drug pharmacology. On the other hand, both M5 and M6 are more than 10 times less potent than dasatinib and are considered minor metabolites circulating in the body. M20 and M24 have reasonable potency [6].

Dasatinib is excreted extensively through feces (around 85%) rather than the renal system, with a half-life of 3–4 h [2,3,4].

CYP3A4 inducers and inhibitors have a major impact on peak plasma concentration (C_max_) and AUC of dasatinib, such as (ketoconazole, a strong CYP3A4 inhibitor and rifampin, a CYP3A4 inducer) [3,4]. Gastric acid modifying agents also can affect the absorption of dasatinib because of its basic nature by affecting the solubilization and this will affect C_max_ and AUC accordingly [4]. Yoshitsugu et al. (2012) reported an approximately 26% increase in C_max_ of dasatinib was observed when Maalox^®^ antacid (aluminum hydroxide and magnesium hydroxide) was administered 2 h before dasatinib administration, while 58% and 55% decreases in C_max_ and AUC, respectively, were reported when Maalox^®^ was co-administered with dasatinib. Famotidine also caused decreases in C_max_ and AUC of dasatinib of 63% and 61%, respectively, when administered 10 h prior to dasatinib administration [7].

The side effects that may occur from TKIs are massive and may extend to all the body systems and organs. Table 1 shows a group of adverse events that were caused by dasatinib.

After dasatinib’s approval and post-marketing, some adverse events were reported, including hepatitis B virus reactivation, interstitial lung disease and nephrotic syndrome [4]. Dasatinib, like all other TKIs, has a relatively high percentage of treatment termination or discontinuation due to adverse events. However, dasatinib is tolerated by both adults and children [3].

Dasatinib is classified as a drug with high pharmacokinetic/pharmacodynamic variability; it has large variabilities (between subject and inter-occasion) of 32–118% and these might be attributed to drug absorption and bioavailability [2,4]. Demetri et al. (2009) studied the pharmacokinetics of dasatinib after administration of different oral doses. The presented results indicated a high variability in different pharmacokinetic parameters such as C_max_, AUC, apparent volume of distribution and apparent oral clearance [8]. The sources/causes of these large variabilities of dasatinib are not clearly evaluated.

Many studies were conducted to examine the effect of patient factors on the pharmacokinetics of dasatinib. Kim et al. (2009) concluded that concomitant medications such as CYP inhibitors and inducers are significant covariates [1], while other studies did not end with significant covariates [7,9].

This study aimed to investigate and evaluate the effect of some subject factors on the concentrations of dasatinib, and to explain the sources of variability, to make the selection of an appropriate dosing strategy for each individual easier in the future.

## 2. Results

### 2.1. Base Model Selection and Full Multi-Covariate Model

A total of 116 healthy subjects were selected to participate in the study. All subjects were Middle Eastern males. Many details on the study population demographics (such as age, BMI, smoking habits and others) are shown in Table 2.

A two-compartment model with first order absorption was selected as the best population pharmacokinetic model for dasatinib concentrations. A transit compartment was implemented to describe delay in onset of absorption. A lognormal distribution was used to describe interindividual variability. The inclusion of inter-occasion variability (IOV) terms for all parameters resulted in reduction in the values of BIC. Hence, the selected basic structural model included IOV terms. A combined error model of constant and proportional terms was used to describe residual error according to the following equation:

$$Y=\left(F+\sqrt[{2}]{a^{2}+b^{2}\cdot F}\right)\cdot \varepsilon$$

where *Y* is the observed concentration, *F* is the model predicted concentration using individual parameters, *a* and *b* are constants, and *ε* is a standardized Gaussian random variable.

### 2.2. Final Model

The results of the final model resulted from covariate screening are presented in Table 3. The model provided accurate estimates of population estimated parameters, and interindividual and inter-occasion variability with a RSE% of less than 50%. Body mass index (BMI) index was the only identified covariate associated with the absorption rate constant, according to the following equation:

$$\log\left(Ka\right)=\log\left(0.37\right)-0.85\cdot log(BMI)$$

where Ka is the absorption rate constant in h^−1^, BMI is body mass index in kg/m^2^.

**Figure 3:**
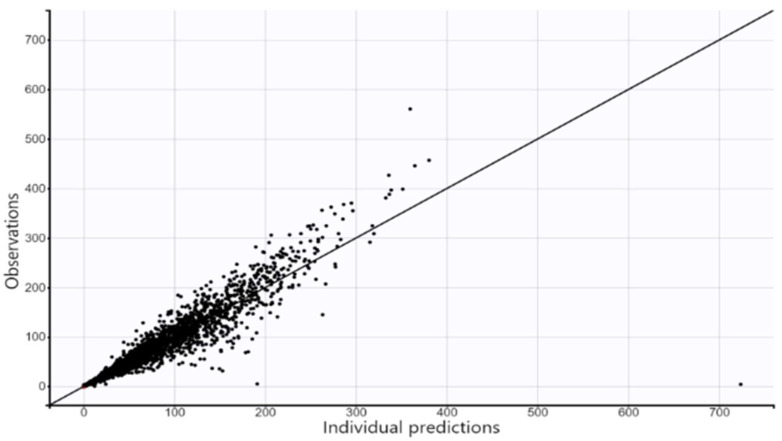
Scatter plot of the observed concentrations (ng/mL) vs. the individual predicted concentrations (ng/mL).

**Figure 4:**
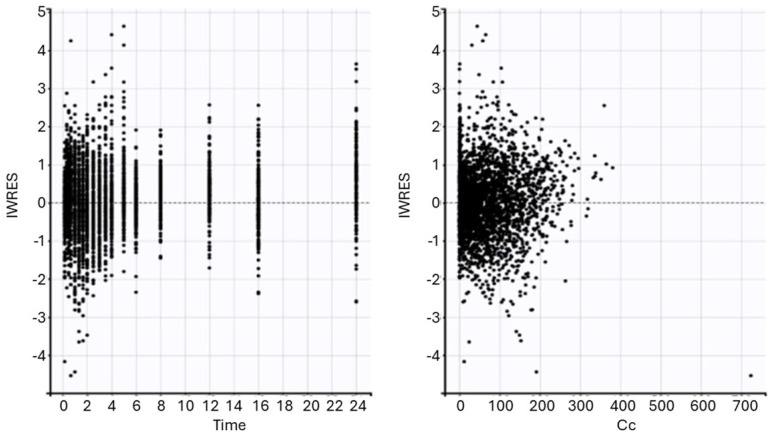
Scatter plot of the individual weighted residuals vs. time and individual weighted residuals vs. the predictions (ng/mL).

The model provided accurate estimates of dasatinib concentrations, as displayed in Figure 3. The predicted vs. observed points were symmetrically distributed around the identity line. Figure 4 displays individual weighted residuals (IWRES) vs. time and (IWRES) vs. predicted concentrations. There was no apparent trend either as a function of time or predicted concentrations. Points were uniformly distributed around an IWRES value of zero.

**Figure 5:**
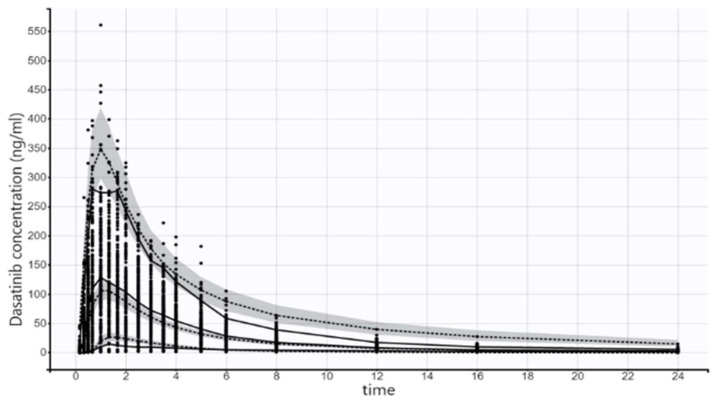
Visual predictive check for the plasma concentrations of dasatinib. The 5th, median and 95th percentile of the observed data are presented as solid black lines while the dashed black lines represent the 5th, median and 95th theoretical percentiles of the simulated data; the shaded gray area represents the 90% prediction intervals.

The predictive performance of the model is displayed in Figure 5. The model predicted values captured the main features of the plasma concentration profile. Empirical and actual medians, the 5th percentile and the 95th percentile were very close. There were few areas where the predicted concentrations tended to be higher than observed concentrations. Variations in the model by changing number of compartments, absorption type and absorption delay did not improve the predictive performance of the model.

**Figure 6:**
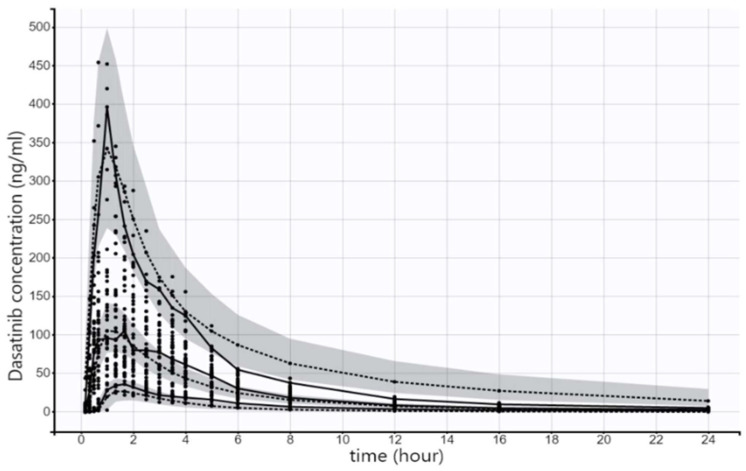
Visual predictive check for the plasma concentrations of dasatinib. The 5th, median and 95th percentile of the observed data of the internal validation dataset are presented as solid black lines while the dashed black lines represent the 5th, median and 95th theoretical percentiles of the simulated data; the shaded gray area represents the 90% prediction intervals.

**Figure 7:**
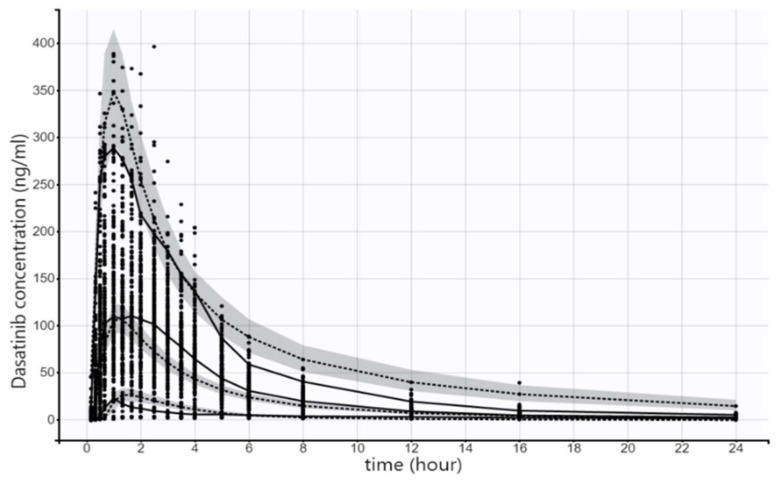
Visual predictive check for the plasma concentrations of dasatinib. The 5th, median and 95th percentile of the observed data of the external validation dataset are presented as solid black lines while the dashed black lines represent the 5th, median and 95th theoretical percentiles of the simulated data; the shaded gray area represents the 90% prediction intervals.

The model also was evaluated using an internal validation dataset, which was 20% of the original analysis dataset (as shown in Figure 6) as well as an external validation dataset, which included data obtained from another bioequivalence study (as shown in Figure 7). In both datasets, the model was deemed to describe the data appropriately.

## 3. Discussion

Dasatinib is well absorbed orally, extensively distributed to the extravascular tissues with large volume of distribution, and extensively metabolized by the liver enzymes, which suggests that hepatic metabolism is the main elimination pathway. Minimal renal excretion and relatively extensive fecal excretion are reported [4].

The two-compartmental model with first order absorption and transit compartment was fitted to the plasma concentrations from 88 subjects (representing the 80% remaining from the whole data after excluding 20% for the internal validation) who were administered dasatinib orally twice separately. This separate dual administration raises the ability to investigate the IOV. The IOV was included because this model gave the smallest BICc value.

Many covariates were tested, such as subject demographics (age; body weight; body mass index; smoking status), laboratory tests (total bilirubin, ALP, AST, ALT, Cr and BUN, glucose, white blood cell count, red blood cells count, platelets count, neutrophils, lymphocytes and hemoglobin level); and concurrent medications (such as paracetamol and diclofenac).

The COSSAC procedure was used as an estimation procedure throughout the whole analysis, which selects the covariate based on its correlation to the individual parameter and is considered more efficient in the number of calculation runs.

The resultant final parameter estimates of some model parameters were comparable with the published ones and some were not, which might be attributed to different physiology between CML patients and healthy subjects [9].

Body mass index was the only tested covariate that showed a significant drop in BICc value when added on Ka.

Body mass index is one of the commonly used describers of body size/obesity. There are many other describers such as lean body weight (LBW), fat-free mass (FFM), ideal body weight (IBW), total body weight (TBW) and adjusted body weight (ABW) [10].

The BMI is calculated by the division of weight (in kilograms) over the height (in meters squared) and classifies the person as underweight, normal weight, overweight, or obese as follows [11]:

Underweight—BMI < 18.5 kg/m^2^;
Normal weight—BMI ≥ to 18.5 to 24.9 kg/m^2^;
Overweight—BMI ≥ to 25 to 29.9 kg/m^2^;
Obesity—BMI ≥ to 30 kg/m^2^.

The chronic inflammation state of adipose tissue, which is also called obesity, is one of the risk factors which potentiate many health problems such as heart diseases, elevated blood pressure, abnormal cholesterol levels and diabetes. The huge effect of obesity on health arises from many factors, such as cardiac output, glomerular filtration, liver blood flow and others.

Obesity can also accelerate gastric emptying and enhance the gastrointestinal permeability to cover for the extra fatty tissue. This effect will correlate to the drug absorption.

The absorption process can be described by the time needed to reach the peak concertation (T_max_) and C_max_ for its rate and by AUC for its extent. The absorption rate constant (Ka) is a value that describes the absorption rate. The effect of obesity on absorption is not definite for all drugs. Generally, obesity/overweight does not affect oral absorption directly while it affects distribution, metabolism and elimination [12].

T_max_ is the time in which the absorption rate equals the elimination rate and it can be calculated as per the equation below:
T_max_ = ln(Ka/K) Ka − K;
T_max_: time to reach peak concentrations;
Ka: absorption rate constant;
K: elimination rate constant.

As per the above equation, any change of Ka or K in a time will lead to a change in t_max_, such as renal impairment, a medical condition which affects elimination. The elimination rate constant will have small value, which results in a higher value for t_max_ and delay in the onset of action [13].

In the study, about 35% of the subjects were considered overweight/obese (BMI more than 25 kg/m^2^); it was observed that the higher value of BMI, the lower the Ka value.

Many benzodiazepines such as diazepam, alprazolam and others have an increase in half-life because of obesity; diazepam’s half-life increase of up to 95 h will lead to a decrease in K and increase in Ka at t_max._ This effect illustrates the dose adjustments made for benzodiazepines in obese patients [14]. Dosing of many other drugs can also be adjusted by obesity, such as some antibiotics, antifungals, heparins and sedatives [15]. The same can be applied for dasatinib based on the study results.

## 4. Methodology and Study Design

### 4.1. Overall Study Design Description

A total of 110 subjects were used in the analysis (88 subjects were used in modeling and 22 subjects were used for internal validation), the data of these subjects were taken from a four-period full replicate bioequivalence study. IRB approval was given on 17 March 2020 and JFDA approval was given on 11 June 2020 in the letter of reference number 2/1/8/18905 (in two periods, the subjects were adminsitered the investigational/test product and in the other two periods, they were adminsitered the originator/reference product accroding to a random scheme). A total of 116 healthy subjects were selected to participate in the study while 110 subjects completed the study. This relatively large number of subjects were expected to provide diverse data, giving the model a better ability to describe the drug behavior correctly.

This planned number of subjects was considered based on the intra-subject coefficient of variation of a previous study, which was around 74%, with a study power of 80% and ratio of 90% and 20% rate of withdrawal.

As per the study design, in two periods of the study, the subjects were administered an oral dose of SPRYCEL^®^ 140 mg film coated tablet, the reference product, under fasting state with 240 mL of water in the morning (one tablet in each period/occasion). No food was allowed until 4 h after dosing. The study was carried out in the International Pharmaceutical Research Center (IPRC) and in accordance with the Declaration of Helsinki and after obtaining approval from the related health and subjects rights committees (Jordan Food and Drug Administration (JFDA) and Instituational Review Board (IRB)). Each subject also received a copy of an informed consent document (ICD), in which all study details, especially those related to subject safety, were presented.

The subjects who met the eligibilty criertia were enrolled. Inclusion and exclsuion crieria are listed below:

- Inclusion criteria
  1. Age 18 to 55 years, inclusive.
  2. Body mass index (BMI) range is within 18.5–30.0 Kg/m^2^.
  3. Subject does not have a known allergy to the drug under investigation or any of its ingredients or any other related drugs.
  4. Medical history and physical examination within medically acceptable criteria.
  5. Standard 12-lead ECG assessment is normal.
  6. Males and their female partners need to practice adequate contraception for at least one week after the dasatinib dose.
  7. Laboratory investigation tests within laboratory reference ranges (ALP and creatinine are accepted if below the reference range). Hematology tests within 5% of reference limits.
- Exclusion criteria
  1. Medical demographics performed not longer than two weeks before the initiation of the clinical study with significant deviations from the normal ranges.
  2. Presence of any clinically significant results from laboratory tests; however, ALP and creatinine will be accepted if below reference range. Hematology tests with deviation of more than 5% of the reference limits. Laboratory tests are performed not longer than two weeks before the initiation of the clinical study.
  3. History of drug or alcohol abuse.
  4. Subject is a heavy smoker (more than 10 cigarettes per day).
  5. Positive drug abuse test screening and/or positive alcohol test at screening.
  6. Subject does not agree not to take any prescription or non-prescription drugs with systemic absorption within at least two weeks preceding the first study drug administration until donating the last sample of the study.
  7. Subject does not agree not to take any vitamins for nutritional purposes within at least two days before first study drug administration until donating the last sample of the study.
  8. Subject is on a special diet (for example, subject is a vegetarian).
  9. Subject consumes large quantities of alcohol or beverages containing methylxanthines, e.g., caffeine (coffee, tea, cola, energy drinks, chocolate, etc).
  10. Subject does not agree not to consume any beverages or food containing alcohol at least 14 days prior to first study drug administration until donating the last sample of the study.
  11. Subject does not agree not to consume any beverages or food containing methylxanthines, e.g., caffeine (coffee, tea, cola, chocolate, etc.) at least 24 h prior to the study drug administration of each study period until the end of confinement period.
  12. Subject does not agree not to consume any beverages or food containing grapefruit at least two weeks prior to first study drug administration until donating the last sample in the study.
  13. Subject has a history of severe diseases which have direct impact on the study.
  14. Participation in a bioequivalence study or in a clinical study within the last 80 days before first study drug administration.
  15. Subject intends to be hospitalized within 3 months after first study drugs’ administration.
  16. Subject donated blood or its derivatives in the past 3 months or who through completion of this study, would have donated more than 1250 mL in 120 days, 1500 mL in 180 days, 2000 mL in 270 days, 2500 mL of blood in 1 year.
  17. A positive pregnancy test or subject is lactating during screening or study period if the subject is female.
  18. Subject has a history of significant asthma, peptic or gastric ulcer, sinusitis, pharyngitis, renal disorder (impaired renal function), hepatic disorder (impaired hepatic function), cardiovascular disorder, neurological disease such as epilepsy, hematological disorders or diabetes, psychiatric, cardiopulmonary disease, congenital long QT syndrome, fluid retention, dermatologic or immunological disorders.
  19. Subject does not agree not to engage in strenuous exercise at least one day prior to study drug administration until donating the last sample in each respective period.
  20. Subject having at screening examination a pulse outside the normal range of (60–100 beats per minute) or a body temperature outside the normal range of (35.0–37.2 °C) or a respiratory rate outside the normal range of (14–20 breaths per minute) or a sitting blood pressure less than 100/60 mm Hg or more than or equal to 140/90 mm Hg.
  21. Subject has history of difficulties in swallowing or any gastrointestinal disease which could affect the drug absorption.
  22. Subject does not agree not to consume any medication or food which may affect CYP3A4 enzyme at least two weeks prior to first study drug administration until donating the last sample of the study.
  23. Subject has history of malabsorption within the past year or presence of clinically significant gastrointestinal disease.
  24. Subject has galactose or fructose intolerance, sucrase-isomaltase insufficiency, Lapp lactase insufficiency, galactosemia, or glucose–galactose malabsorption syndrome.
  25. Subject has a history of hypokalemia or hypomagnesemia.

We drew 19 blood samples (5 mL) using an arm-inserted cannula as per the below timing schedule: before dosing (0.00 h) and after the dosing at 0.167 (10 min), 0.333 (20 min), 0.50 (30 min), 0.667 (40 min), 1.00, 1.33 (1 h and 20 min), 1.67 (1 h and 40 min), 2.00, 2.50, 3.00, 3.50, 4.00, 5.00, 6.00, 8.00, 12.00, 16.00 and 24.00 h.

The samples were collected in tubes containing tri-potassium ethylenediaminetetra-acetic acid (K_3_EDTA) as an anticoagulant. Samples then were centrifuged to obtain the plasma, which was analyzed for dasatinib concentrations. The plasma samples that resulted from centrifugation of blood samples were stored at a temperature of −20 °C till analyzed. Vital signs (blood pressure, temperature, heart rate and repiratory rate) were evaluated during the course of the study; also adverse events reported by the subjects were recorded and appropriate treatments were given.

### 4.2. Bioanalysis

A selective, sensitive and rapid liquid chromatography–tandem mass spectrometry (LC/MS/MS) method for the determination of dasatinib concentrations in plasma was validated. The procedure involved liquid–liquid extraction of dasatinib and the use of labeled internal standard (dasatinib-d8).

The chromatographic separation employed a C8 column (100 × 4.6 mm); the mobile phase consisted of ammonium acetate buffer, formic acid and acetonitrile. Detection was carried out using a triple quad mass spectrometer (Mass Detector API 4000, Applied Biosystem-SCIEX-MDS, London, ON, Canada) in multiple reaction monitoring (MRM) mode using turbo ion spray with positive ionization.

The method was validated considering different parameters including assay recovery, specificity, linearity, accuracy, precision, sensitivity and stability. The method had a total run time of about 4 min and showed acceptable linearity (r > 0.99) over the working range of 0.50–500.00 ng/mL.

### 4.3. Population Pharmacokinetic Modeling

Population pharmacokinetic modeling was performed using nonlinear mixed-effect modeling software (Monolix version 2020R1. Antony, France: Lixoft SAS, 2019. http://lixoft.com/products/monolix/) accessed on 9 December 2020.

#### 4.3.1. Base Model

Several scenarios for the base model were examined using Monolix (version 2020R1). This stage specified the basic structural model features including number of compartments, absorption delay model, error model, interindividual variability and inter-occasion variability. The explored number of compartments was one, two and three compartments. Two absorption delay models, lag time and transit delay compartment, were also compared to the absorption without delay model. Following the determination of the basic structural model, the influence of inclusion of inter-occasion variability was also explored. Base model selection was based on the value on Bayesian information criterion (BIC), goodness of fit diagnostic plots, and relative standard errors (RSE) of the estimated parameters [16].

A lognormal distribution was assumed for individual parameters. Interindividual and inter-occasion variability and possible covariate relationships were included according to the following equation:

$$\log\left(\theta_{i}\right)=log\left(\theta \right)+\beta \cdot Cov+\eta_{i}+\eta_{ik}$$

where $\theta_{i}$ is the individual pharmacokinetic parameter, θ is the population pharmacokinetic parameter, β is the covariate regression term, ${Cov}_{i}$ is the covariate for the ith individual, $\eta_{i}$ is the random effect for the ith individual and $\eta_{ki}$ is the random effect for the ith individual at the kth occasion. The random effects $\eta_{i}$ and $\eta_{ki}$ were assumed to follow normal distributions $\eta_{i}\sim N(0,\omega )$ and $\eta_{ki}\sim N\left(0,\gamma \right),$ where ω and γ are standard deviations of the interindividual and inter-occasion variability terms.

#### 4.3.2. Building the Full Multi-Covariate Model

Explored subject characters examined as potential covariates included age; body weight; body mass index; smoking status (smoker subject was a subject who smoked more than 10 cigarettes per day); liver function tests including total bilirubin, alkaline phosphatase (ALP), aspartate aminotransferase (AST), alanine aminotransferase (ALT); kidney function tests including creatinine (Cr) and blood urea nitrogen (BUN); glucose; blood indices including white blood cell count, red blood cell count, platelet count, neutrophils, lymphocytes and hemoglobin level; and concurrent medications (such as paracetamol and diclofenac).

Conditional sampling for the stepwise approach based on correlation tests (COSSAC) and a covariate screening process was implemented [17]. COSSAC is a Monolix-based automatic covariate screening procedure. Model selection with COSSAC was based on a statistically significant difference in the corrected Bayesian information criteria (BICc) at a level of significance during forward selection and at a level of significance of *p* = 0.01 during backward selection step.

#### 4.3.3. Model Evaluation

A visual predictive check (VPC) was used to evaluate the predictive performance of the final model. The final population pharmacokinetic model was used to generate 1000 simulated datasets for the population. The simulated dataset was used to plot the VPC. The 5th, 50th and 95th percentiles of the simulated concentrations were constructed and compared against the observed concentrations. VPC assesses the predictive performance of the model.

VPC was applied to three different sets of data. The first dataset was the data used for model development and estimation of pharmacokinetic parameters. The second dataset represented an internal validation dataset, which represented 20% of the analysis dataset taken apart prior to the analysis and then used as a validation dataset to evaluate the predictive performance of the final model internally. A third dataset composed of data of 90 subjects from another pharmacokinetic study, JFDA approval letter number 2/1/8/43262, was used to externally validate the final model.

## 5. Conclusions

The main goal from population pharmacokinetic studies is to know what is/are the factor/s to be considered for dosing determination.

One of the main concerns in health is how to ensure that the dose taken by the patient leads to an effective drug exposure and therapy. Many patients might complain of side effects (because of supra-therapeutic effects) or feeling unimproved (because of sub-therapeutic effects), resulting from the lack of knowledge of factors that might affect the dosing regimens.

Dasatinib, a promising agent for leukemia patients and in some cases with a high benefit to risk ratio, should be carefully prescribed.

The variability of dasatinib might be determined by the patient’s body mass index. Taking this factor into consideration might be helpful in the future to determine the correct dose for each individual or to modify the dose (dose enlargement or dose reduction) in order to ensure that the effective concentrations are reached for that patient. However, the study had some limitations, including that the data used for analysis were taken from healthy volunteers, who rendered the data of many covariates within the reference ranges, which is not the case in the real patients. In the future studies, it is recommended to perform this analysis on a patient population (the target population that will take the drug) in order to take their factors into account in the analysis; also race and gender factors could not be studied because the participating subjects in the study were Middle Eastern males only.

Modeling itself had some limitations, such as the assumption of the data distribution. In this study, log distribution was assumed for the individual parameters within the population, which may not have fully captured the underlying variability in drug metabolism and distribution; also the choice of model structure depended on the availability of data to support model estimation.

It is recommended to perform more studies on different doses and research on this drug and the other TKIs, since these might give promising results if appropriate concentrations are reached. Enrollment of patients with abnormal hepatic/renal function might be an important issue to be studied.

## Figures and Tables

**Table 1:** Adverse events reported by CML and ALL patients (newly diagnosed and resistant CML) treated by dasatinib (as monotherapy or combination) [4].

System/Organ	Adverse Events
Blood and vascular system	Thrombocytopenia ^a^, neutropenia ^a^, bleedings ^b,c^
Respiratory system	Pleural effusions, dyspnea, dry cough, chest pain, pulmonary arterial hypertension ^a^, pulmonary edema
Cardiovascular system	Ischemia, palpitations, arrhythmias, QT Prolongation ^d^, congestive heart failure and cardiac dysfunction
Skin	Rash, Stevens–Johnson syndrome (SJS) and erythema multiforme, pruritus
Nervous system	headache
Gastrointestinal system	Diarrhea, abdominal pain, nausea, vomiting, constipation and mucositis
Musculoskeletal system	Myalgia, arthralgia, muscle spasms
Infections	Bacterial, viral and fungal infections
General	Decreased appetite, fluid retention and pain

^a^: Leads to treatment discontinuation; ^b^: might be fatal; ^c^: mainly in the GIT and might happen in the CNS; ^d^: patients with hypokalemia and hypocalcaemia should not take dasatinib.

**Table 2:** Demographics of the study population.

Characteristic	(Mean ± Standard Deviation (SD))	Median (Range)
Gender	Males	NA
Count	116	NA
Age (Years)	32 ± 8.36	33 (18–49)
Body weight (Kg)	72 ± 12.71	70 (51–100)
Height (cm)	175 ± 5.86	175 (160–192)
BMI (Kg/m^2^)	23.6 ± 3.50	22.9 (18.6–29.8)
Smoking history *	14.66% were non-smokers, 85.34% were smokers	NA
Race	Middle Eastern	NA

*: Data demonstrated as percentage; NA: not applicable.

**Table 3:** Summary of final model parameters.

Parameter	Description	Population Parameter (%RSE)	Between-Subject Variability (Standard Deviation (%RSE))	Inter-Occasion Variability (Standard Deviation)
Ktr	Transit time rate constant	18.8 (9.34)	0.49 (26)	0.84 (9.54)
Mtt	Meant transit time	0.48 (4.63)	0.3 (16.1)	0.4 (8.04)
Ka	Absorption rate constant	0.37 (4.8)	0.36 (12.7)	0.31 (10.1)
β_BMI_	Regression coefficient for the effect of body mass index (BMI) on Absorption rate constant	−0.85 (36.9)		
Cl	Clearance	273.14 (7.37)	0.62 (9.82)	0.42 (7.94)
V1	Volume of the central compartment (L)	18.98 (8.94)		
Q	Intercompartmental clearance (L/h)	64.62 (3.92)		
V2	Volume of the peripheral compartment (L)	487.9 (3.18)		
a (constant)	Residual variability	0.78 (5)		
b (proportional)	Residual variability	0.22 (1.72)		

%RSE is the percentage relative standard error.

## Data Availability

Data are available on request from the corresponding author.
